# Investigating the influence of perinatal fluoxetine exposure on murine gut microbial communities during pregnancy and lactation

**DOI:** 10.1038/s41598-024-62224-7

**Published:** 2024-06-14

**Authors:** Katelyn Desorcy-Scherer, Ibrahim Zuniga-Chaves, Maggie A. Reisner, Garret Suen, Laura L. Hernandez

**Affiliations:** 1 School of Nursing, University of Wisconsin-Madison, 701 Highland Avenue, Madison, WI 54705 USA; 2 Department of Bacteriology, University of Wisconsin-Madison, Madison, WI USA; 3 Microbiology Doctoral Training Program, University of Wisconsin-Madison, Madison, WI USA; 4 Department of Animal and Dairy Sciences, University of Wisconsin-Madison, Madison, WI USA

**Keywords:** Gut microbiome, Perinatal, Antidepressant, Maternal, Infant, Microbiology, Health care

## Abstract

Selective Serotonin Reuptake Inhibitor (SSRI) therapy is common among perinatal populations for the treatment of mood disorders. Medications can affect diversity and composition of the gut microbiome, which plays a key role in modulating health. While previous studies have examined the effects of antidepressant exposure on the maternal gut microbiome, whether SSRI exposure affects the offspring gut microbiome is unknown. We investigated the effects of maternal fluoxetine exposure on the gut microbiome of maternal and offspring mice during pregnancy and lactation (embryonic day 10–lactation day 21; E10–L21). Stool samples collected on E17, L11, L15, and L21 were examined using 16S rRNA sequencing. Our results suggest that maternal fluoxetine exposure may result in decreased alpha diversity of the offspring gut microbiome in early life. Furthermore, we observed several genera-specific differences in the gut microbiome based on treatment, specifically of *Turicibacter*, *Parasutterella*, and *Romboutsia*. These findings support our understanding of gut health, as dysbiotic development of the gut microbiome has been associated with local and systemic health problems including gastrointestinal morbidities and interrupted growth patterns in infants. Future research should pursue study in human populations and those at high risk for gut microbial dysbiosis and intestinal injury.

## Introduction

Perinatal mental health disorders occurring during pregnancy and up to 12 months following delivery affect up to 20% of the population and represent one of the most common complications of pregnancy and postpartum^1^. Those with mental health disorders pre-gestation are at particularly high risk for experiencing a disorder in the pregnancy or postpartum period^2^. The burden and cost to the U.S. heathcare system is immense, with recent estimates suggesting that mental health disorders increase severe maternal morbidity by up to 50%, with delivery-related hospitalization costs in excess of $102 million dollars annually^3,4^. Recently, the Joint Commission declared that the United States is facing a maternal health crisis and listed maternal mental health conditions as a leading cause of pregnancy-related death^5^.

Approximately 5–13% of mothers receive treatment with a common class of antidepressants, selective serotonin reuptake inhibitors (SSRIs). Estimates suggest that the highest rates of SSRI exposure worldwide occur in the United States^6–8^. Although many medications exist within the SSRI class, sertraline (Zoloft®), citalopram (Celexa®) and fluoxetine (Prozac®) are among the most frequently prescribed SSRIs in perinatal populations^9^. Like many other medications, SSRIs can cross the blood-placental and blood-milk barrier to reach both fetal circulation and human milk^10^. The decision to initiate or continue treatment with SSRIs during pregnancy requires consideration of risk to benefit and can be complicated by the sparsity of research on how perinatal SSRI exposure might affect short and long-term health outcomes.

Antidepressants of the SSRI class are thought to act by manipulating serotonin signaling in the central nervous system, although oral administration may result in greater systemic effects^11^. In fact, SSRIs have antimicrobial properties which have sparked growing interest in the relationship between antidepressant treatment, gut serotonin bioavailability and the gut microbiome^12,13^. Disruptions to serotonin homeostasis through antidepressant exposure have been shown to affect the fitness of the resident gut microorganisms^14,15^. In turn, members of the gut microbiome can modulate peripheral serotonin production, likely through the secretion of metabolites^14^. The competing influences between antidepressant exposure, gut serotonin dynamics and microbial activity illustrate the critical yet complex role of serotonin in the gut microbial ecosystem and raises further questions about the the gut microbiome’s role in mental health.

While accumulating evidence links depression and SSRI antidepressant exposure to gut microbial composition, the specific nature of influence across populations may vary^16,17^. Research conducted with adult nulliparous participants affected by major depressive disorder suggests that antidepressant exposure can decrease the Firmicutes/Bacteriodetes ratio and negatively impact alpha diversity^18^. At higher resolution, reductions in the *Turicibacter* genre have also been described^19^. Despite the burden of maternal mental health conditions during perinatality, less is known about the effects of maternal antidepressant exposure on the gut microbiome in mothers and offspring. Study of perinatal rodent models suggest SSRI exposure could potentially decrease maternal populations of Bacteroides while increasing *Lachnospiraceae*, *Lachnoclostridium* and *Blautia*^20,21^. Consideration of antidepressant effects on the gut microbiome of perinatal populations is crucial, as it’s possible that effects may be unique or potentially compounded by gut microbiome plasticity or transitions that occur during pregnancy and lactation^20,21^.

Although our understanding surrounding maternal antidepressant exposure and the gut microbiome is rapidly expanding, whether maternal antidepressant exposure affects early infant gut microbiome diversity and composition has not been explored. The infant gut microbiome is shaped by a variety of social, cultural and environmental determiants and ultimately, may be related to both short and long-term health^22–24^. Here, we sought to address this important gap in our knowledge by examining the effect of fluoxetine (SSRI) exposure on the maternal and offspring gut microbiome in a rodent model.

## Results

In cohort one, 20 female mice were ordered and 11/20 completed the model of pregnancy. Six were randomized to the fluoxetine (treatment) group and five were randomized to the saline (control) group. During the experimental period, a total of 53 stool samples were collected, sequenced, and used in the analysis (E10 = 9, E17 = 11, L11 (maternal) = 11, L11 (offspring) = 22). In cohort two, 12/40 mice completed the model of pregnancy. Five were randomized to the fluoxetine group and 7 were randomized to the saline group. A total of 79 samples were collected, sequenced and used in the analysis (E10 = 12, E17 = 12, L15 (maternal) = 12, L15 (offspring) = 10, L21 (maternal) = 12, L21 (offspring) = 21). Six negative control samples were sequenced during DNA extraction and sequencing procedures, for a grand total of 138 sequenced samples.

A total of 3,535,190 reads were sequenced with an average of 25,617+/− 64,612 reads per sample. After processing through QIIME II, our dataset included 1,742 taxa, with 13 taxa that were removed during the decontam procedure^25^. After removal of negative controls and samples with read counts below 5,000, a total of 114 samples remained. Of the 17 samples removed for low read counts, 4 were maternal (2 = E10, 1 = E17, 1 = L11) and 14 were offspring (11 = L11, 2 = L15, 1 = L21). Following rarefaction of the data, 331 ASVs were removed resulting in a final dataset of 1,389 taxa and 114 samples. One sample (offspring L15) was removed from the dataset as an outlier following alpha diversity analyses.

### Alpha diversity

**Figure 1:**
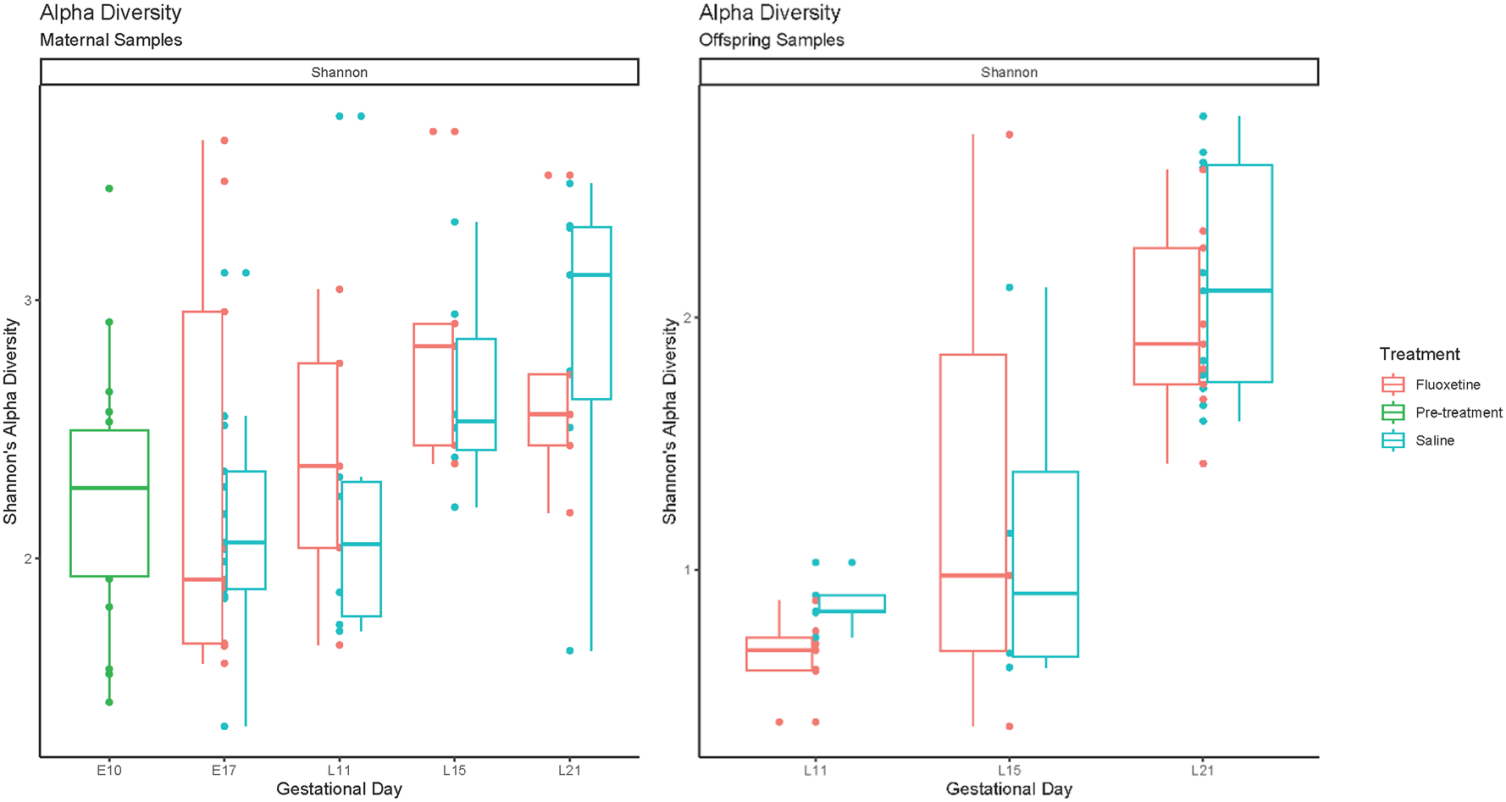
Maternal and offspring alpha diversity between treatment groups. Alpha Diversity was calculated with Shannon’s Alpha Diversity Index and visualized across time. Associated comparisons were calculated with Wilcoxon Signed Rank Test (Table 1). The offspring Alpha Diversity comparison at L11 is statistically significant with* p* = 0.03. E10 = embryonic day 10, E17 = embryonic day 17, L11 = lactation day 11, L15 = lactation day 15, L21 = lactation day 21.

Maternal and offspring populations displayed distinct patterns in the progression of gut microbial alpha diversity over time. In maternal rodents that received either treatment (fluoxetine) or control (saline) exposures, alpha diversity appeared to trend upwards as gestation and lactation progressed. Upon comparison, there were no statistically significant differences in alpha diversity of the maternal gut microbiome in treatment and control mice. In contrast, the offspring of mothers treated with fluoxetine had lower alpha diversity values than the offspring of mothers treated with saline at L11 (*p* = 0.03), with increasing alpha diversity and similarity observed with time (Table 1, Fig. 1).

**Table 1:** Comparison of maternal and offspring alpha diversity across treatment group.

	Maternal	Offspring
E17	0.74	–
L11	0.66	0.03*
L15	0.79	0.99
L21	0.53	0.50

E17 Embryonic day 17; L11 Lactation day 11; L15 Lactation day 15; L21 Lactation day 21. Comparisons made with Wilcoxon Signed Rank Test between treatment groups (fluoxetine/saline). Data listed are *p* values.

*Denotes statistical significance. Values calculated with Shannon’s Alpha Diversity Index.

### Beta diversity

**Figure 2:**
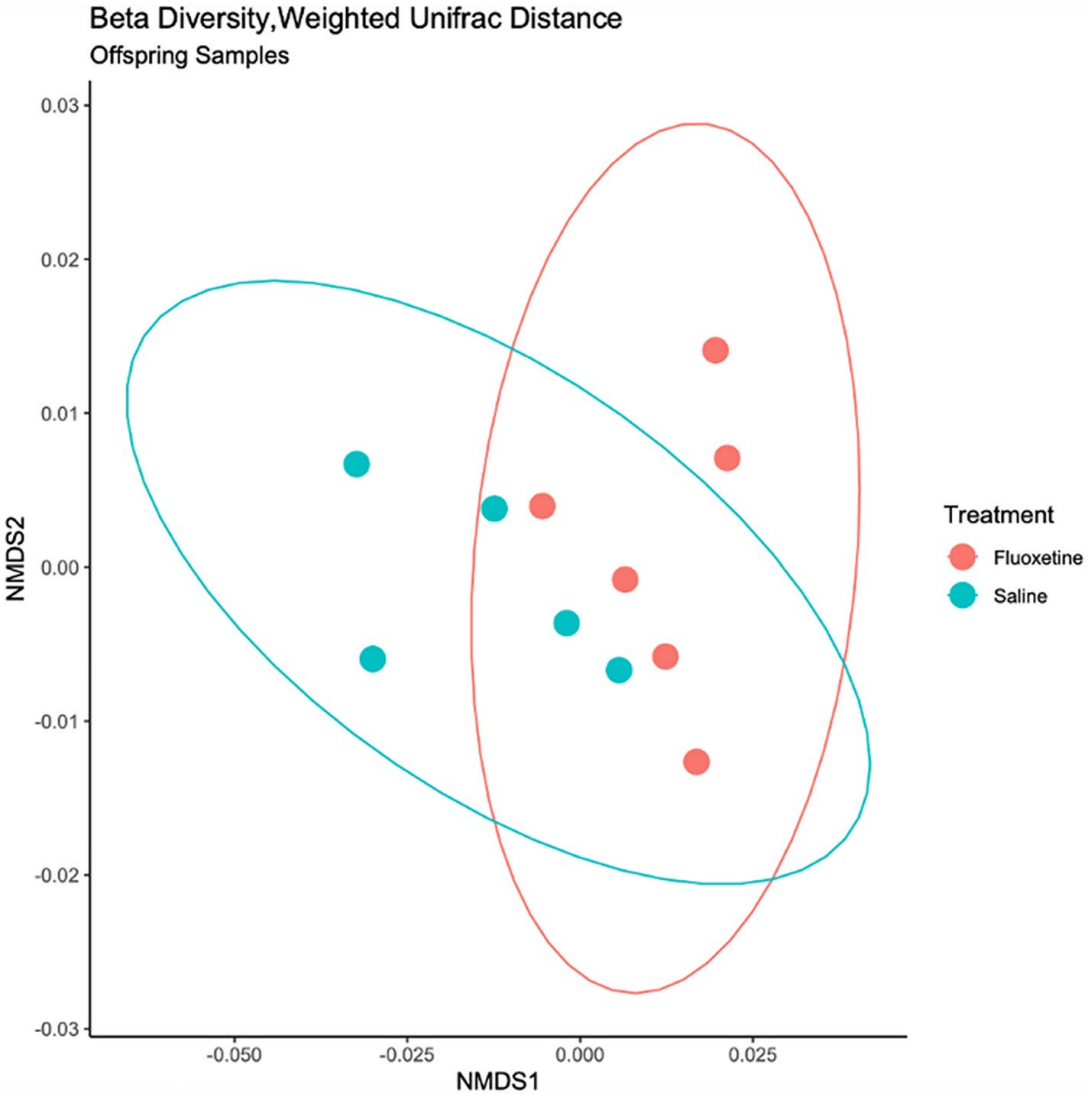
Offspring beta diversity at lactation day 11 between treatment groups. Beta Diversity was calculated with Weighted Unifrac distance and visualized through non-metric multidimensional scaling (NMDS) at lactation day 11. Associated values were calculated with PERMANOVA are statistically significant, *p* = 0.01.

Measures of beta diversity were calculated for maternal and offspring populations and compared by treatment group using Weighted Unifrac distances, Unweighted Unifrac distances and the Bray–Curtis index. Beta diversity measures of maternal samples were similar across treatment groups. In contrast, Weighted Unifrac distance and the Bray–Curtis index revealed statistically significant differences between samples from offspring with maternal exposure to fluoxetine or the control group at L11 (*p* = 0.01; *p* = 0.02; Table 2, Figs. 2, S1). Differences in the Bray–Curtis and Weighted Unifrac measures between treatment groups of offspring at L11 suggest that the relative abundances of microorganisms between treatment groups may be responsible for the observed differences, rather than presence or absence of unique taxa.

**Table 2:** Comparison of maternal and offspring beta diversity across treatment groups.

	Maternal *p* value/R^2^ value			Offspring *p* value/R^2^ value		
Distance metric	Unweighted unifrac	Weighted unifrac	Bray curtis	Unweighted unifrac	Weighted unifrac	Bray curtis
E17	0.22/0.06	0.16/0.08	0.20/0.07	–	–	–
L11	0.29/0.11	0.30/0.12	0.28/0.12	0.27/0.10	0.01*/0.42	0.02*/0.34
L15	0.49/0.10	0.14/0.16	0.24/0.12	0.97/0.09	0.40/0.16	0.21/0.16
L21	0.31/0.10	0.76/0.04	0.70/0.07	0.18/0.07	0.15/0.08	0.06/0.09

E17 Embryonic day 17; L11 Lactation day 11; L15 Lactation day 15; L21 Lactation day 21. Comparisons made with PERMANOVA between treatment groups (fluoxetine/saline). Data listed are *p* values.

*Denotes statistical significance.

### Community composition

**Figure 3:**
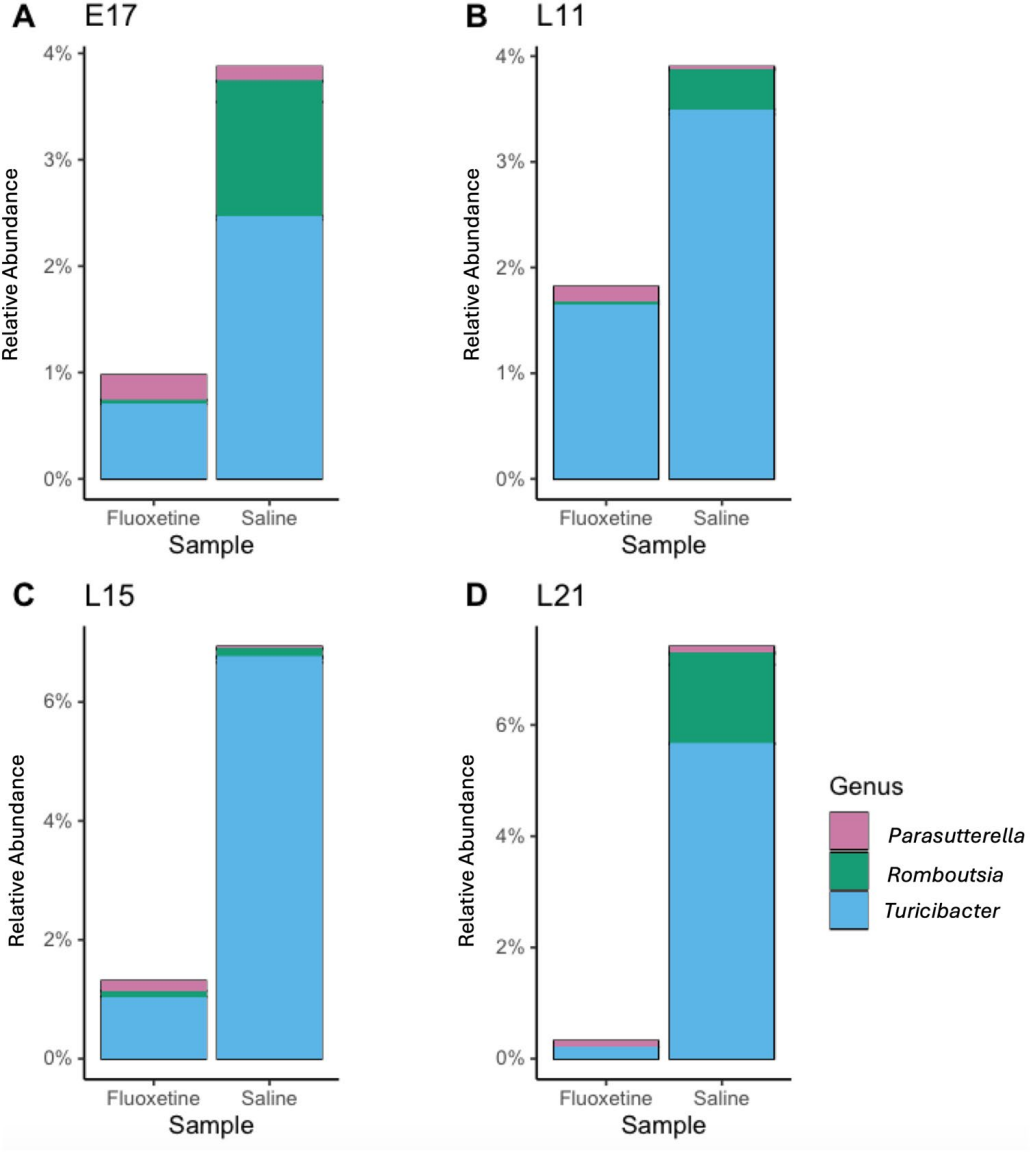
Maternal genera of interest between treatment groups. Relative abundance of *Parasutterella*, *Romboutsia*, and *Turicibacter* at E17, L11, L15 and L21. See Table 3 for inferential tests of statistical significance.

**Figure 4:**
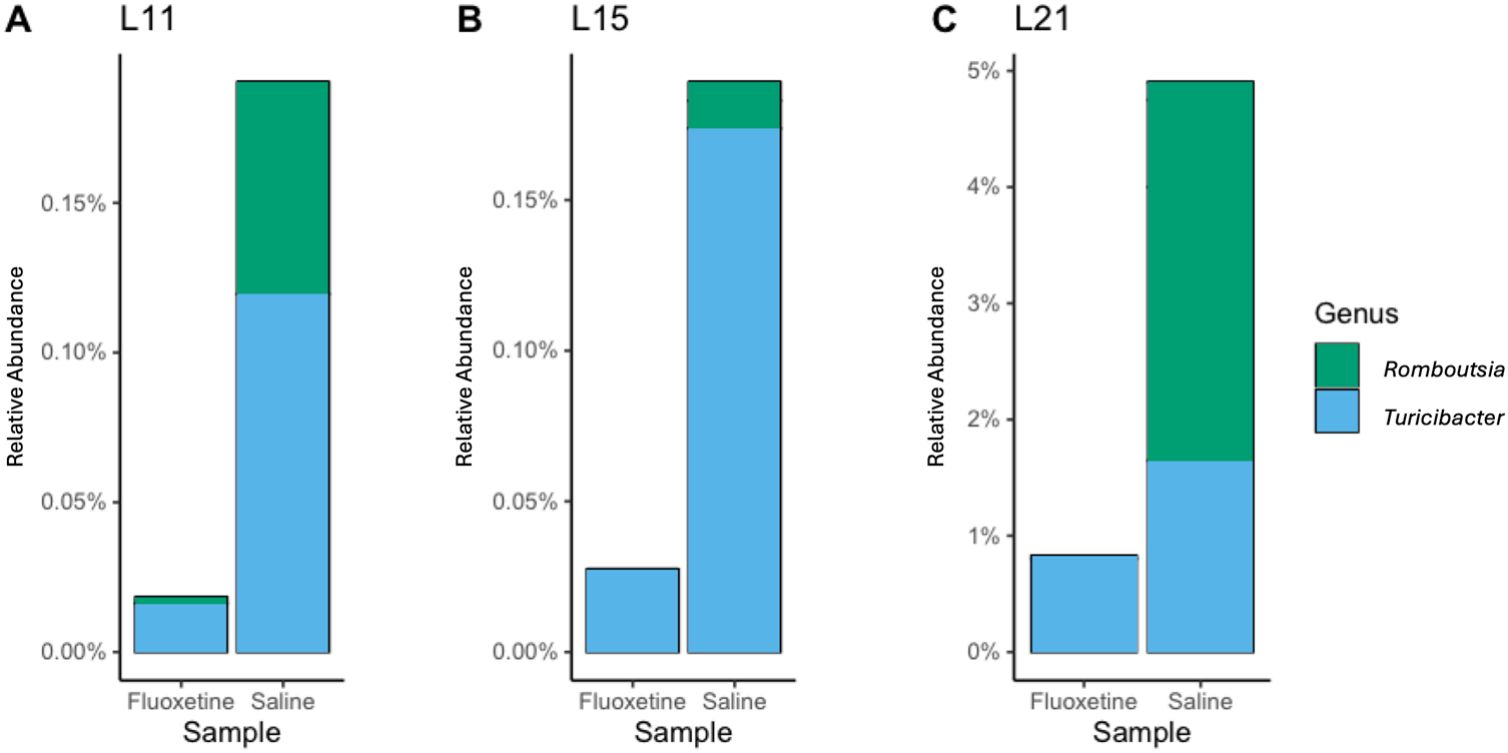
Offspring genera of interest between treatment groups. Relative abundance of *Romboutsia* and *Turicibacter* at L11, L15 and L21. See Table 3 for inferential tests of statistical significance.

Composition of the maternal and offspring gut microbiome was determined using the analysis of compositions of microbiome with bias correction (ANCOM-BC). In maternal samples collected at E17 and L11, there were no significant ASVs found to be different between mothers in the treatment or control groups. However, mothers treated with fluoxetine had higher relative abundances of *Parasutterella* at L15 (*p* = 0.008), and lower relative abundances of *Turicibacter* at L21 (*p* = 0.002; Table 3; Fig. 3). The offspring of mothers treated with fluoxetine had lower relative abundances of *Turicibacter* and *Romboutsia* at L11 (*p* = 0.08; *p* = 0.02; Fig. 4). Similarly, the offspring of mothers treated with fluoxetine had lower relative abundances of *Romboutsia* at L21 (*p* = 0.05).

**Table 3:** Differentially abundant taxa in maternal and offspring samples.

Host	Eday	Genus	Unadjusted *p* value	Adjusted *p* value	Change direction
Maternal	L15	*Parasutterella*	< 0.001	0.008*	↑ in Fluoxetine
Maternal	L21	*Turicibacter*	< 0.001	0.002*	↓ in Fluoxetine
Offspring	L11	*Romboutsia*	< 0.001	0.02*	↓ in Fluoxetine
Offspring	L11	*Turicibacter*	0.002	0.08*	↓ in Fluoxetine
Offspring	L21	*Romboutsia*	< 0.001	0.05*	↓ in Fluoxetine

E17 Embryonic day 17; L11 Lactation day 11; L15 Lactation day 15; L21 Lactation day 21. Comparisons were made in ANCOM-BC. Taxa with adjusted *p* value ≤ 0.1 were considered differentially abundant.

*Denotes statistical significance.

## Discussion

Here we show that maternal fluoxetine exposure may result in decreased offspring alpha diversity in early life, a finding that may be particularly relevant for high-risk infants. Medically complex populations including low birthweight infants often already display lower gut microbial diversity compared to healthy, term-born infants^26^. A low diversity gut microbiome may be less stable than those that are higher and could be more easily perturbed by influences known to affect the infant gut microbiome such as antibiotic exposure^27^. It is also possible that a low diversity gut microbiome may be deficient in certain metabolites produced by missing microorganisms or suffer from a lack of functional redundancy. As such, infants with gut microbiomes deficient in certain metabolites may suffer from increased infection susceptibility and poor postnatal growth^28^. We note that early life growth patterns are particularly important, considering that positive growth patterns are associated with a variety of infant health outcomes, including sufficient neurocognitive development and markers of metabolic health^29^.

Our study also identified associations between maternal fluoxetine exposure and altered abundance of certain bacteria such as those belonging to the genus *Turicibacter*. Species of *Turicibacter* are known to possess a serotonin sensor (CUW_0748) with high structural homology to SERT, a serotonin transporter in mammalian cells that serves as the binding target of SSRI antidepressants^30^. Intestinal conditions high in serotonin have been shown to support the growth of *Turicibacter sanguinis*, but their abundance is significantly depleted by exposure to fluoxetine^15^. Our study supports this finding, as we observed that mothers treated with fluoxetine had lower relative abundance of *Turicibacter* at L21. However, we acknowledge that the decreased abundance we observed may result from other factors such as competitive colonization by other gut microbiota. We also observed similar changes in offspring *Turicibacter* abundance at L11, and our data suggests that *Turicibacter* are higher in abundance in the gut microbiomes of both mothers and offspring without fluoxetine exposure (Figs. 3 and 4).

It is important to note that there is accumulating evidence that supports the role of *Turicibacter* in systemic health. Members of the *Turicibacter genus* have been associated with depression severity and antidepressant exposure in both animal and human populations, and higher abundances of *Turicibacter* have been linked to lower reports of depression symptom severity^31^. This observation may be related to changes in serotonin signaling, as *Turicibacter* have been shown to respond positively to serotonin availability^30^. Gut microbial *Turicibacter* abundance may also serve as a determinant of metabolic health. Studies of *Turicibacte*r *sanguinis* have shown altered intestinal expression of genes related to lipid metabolism and reduced host serum triglyceride levels in monocolonized mice. Moreover, *T. sanguinis* appears to regulate fat mass in sex-specific patterns where only female *T. sanguinis*-colonized mice exhibited reduced mass of inguinal white adipose tissue, relative to males^15^. In human populations, low abundance of *Turicibacter* were observed in children of mothers that were overweight^32^. *Turicibacter* are producers of butyrate and low abundances of this important short-chain fatty acid may represent a health risk, particularly in offspring with medical complications including those born with intestinal underdevelopment or in premature infants with low birth weight^27^.

In addition to *Turicibacter* our study also identifies other gut microbes associated with fluoxetine exposure that are likely related to systemic health. For example, we observed lower relative abundances of  bacteria in the genus *Romboutsia* in offspring of mothers treated with fluoxetine. Human studies of SSRI exposure have found reduced abundances of *Romboutsia* in adults treated with SSRIs and that *Romboutsia* is positively correlated with depression symptom severity^31^. Furthermore, high *Romboutsia* abundance has been repeatedly associated with weight gain in children^33,34^. We also found that perinatal fluoxetine exposure is associated with a decrease in the relative abundance of maternal *Parasutterella*. While *Parasutterella* is commonly observed in the mouse and human gut microbiome, studies have suggested that patterns of increased *Parasuterella* abundance may be related to the development and progression of irritable bowel syndrome^35,36^. *Parasutterella* colonization has also been observed in the context of elevated intestinal inflammatory activity and an abundance of *Parasutterella* may exert effects on local and systemic health through production of succinate, an intermediate metabolite that functions in cross-feeding metabolic pathways^35,36^.

We present this study as the first to investigate the effects of maternal antidepressant exposure on the gut microbiome of offspring and acknowledge that our study is not without a number of limitations. While mouse models provide a level of experimental control not feasible in human studies, mice have the capacity to model depression with varying validity^37^. This study did not include a model of depression, although strong evidence supports the effects of depression on the gut microbiome in human populations^17^. Rather, we present this study as proof of concept that maternal fluoxetine exposure has the potential to affect both the maternal and offspring gut microbiome. Mice in this experiment were also treated with fluoxetine via subcutaneous injection which contrasts the traditional oral route of SSRI administration in humans. The use of subcutaneous injection was necessary to standardize antidepressant exposure in an intervention tolerable to maternal mice but to prevent unintentional offspring exposure. It is possible that the antimicrobial activity of SSRI antidepressants was muted by preventing direct administration of the SSRI into the gastrointestinal tract, as in humans. Furthermore, the period of maternal SSRI exposure in this study was 29 days. Because human populations are counseled to initiate antidepressant therapy for a minimum of several weeks before therapeutic effect is expected, the period of exposure in this experiment could be considered relatively short for the assessment of change in bacterial diversity or composition. It is possible that SSRI therapy may affect the human gut microbiome in ways not observed here, due to the inability to model the length of expected SSRI exposure, perinatal stage, and bacterial lifecycle at a level commensurate to what is experienced by humans.

In this study, we found that maternal antidepressant exposure has the capacity to affect both the maternal and offspring gut microbiome during pregnancy and early life. Fluoxetine exposure affected the relative abundance of several gut microbial genera and was associated with reduced alpha diversity of offspring in the early postnatal period. Changes in the relative abundance of specific bacteria within the gut microbiome may be relevant to the mental, gut, and metabolic health of perinatal populations. Future studies should consider examining the effects of other antidepressant medications commonly prescribed to pregnant and lactating people. Our findings support moving towards inquiry in human populations where impacts of depression on the gut microbiome can be considered alongside perinatal antidepressant exposure. Because concurrent antibiotic exposure or other determinants may act on the gut microbiome alongside SSRIs, researchers may consider inclusion of particularly vulnerable populations at high risk for gut microbial dysbiosis and intestinal morbidities such as preterm infants.

## Methods

### Animals

All experiments were approved by the University of Wisconsin-Madison’s Research Animal Care and Use Committee (RARC) under protocol #A005789-R03-A02 and all study procedures were performed in accordance with institutional and RARC guidelines. Reporting is consistent with The Animal Research: Reporting of In Vivo Experiments (ARRIVE) recommendations^38^. Mice were housed in a controlled environmental vivarium for biological research in the Department of Animal and Dairy Science at the University of Wisconsin-Madison. Wild-type C57BL/6J mice were obtained from Jackson Laboratories (Bar Harbor, ME, USA). Animal facilities were maintained at a temperature of 25 °C and a humidity of 50% to 60% with a 12:12 h light–dark cycle and mice had ad libitum access to both food (LabDiet 5015, TestDiet, Richmond, IN, USA) and water. All animals in our study were fed from the same food lot which was kept separate from food used for other experiments and were euthanized via carbon dioxide followed by cervical dislocation.

### Experimental design

**Figure 5:**
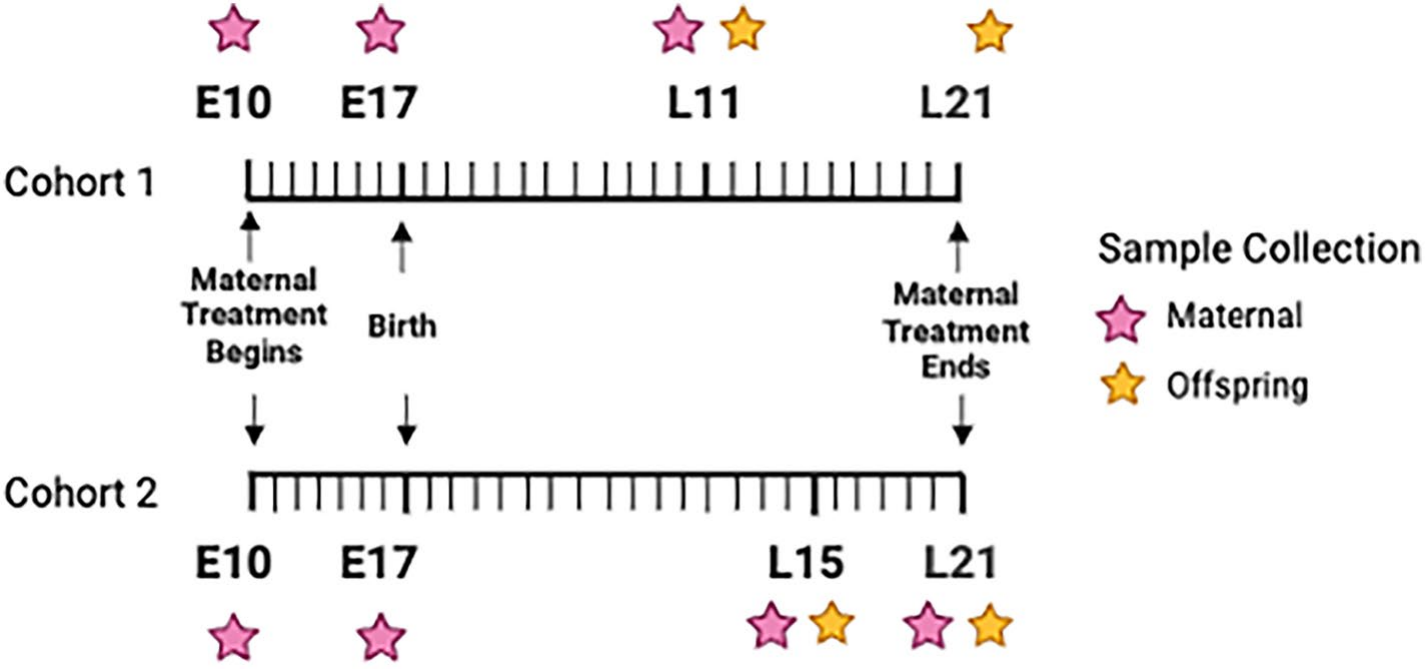
Experimental design. Illustration of the experimental design in Cohort one and Cohort two.

Mice were ordered at four weeks of age and housed according to RARC guidelines (based on weight and sex, up to six mice per cage unit) for two weeks. During this time, we followed a procedure to standardize the starting gut microbiome between animals, which included mixing cage bedding between study animals weekly^39^. We created a perinatal mouse model by breeding female mice with male mice beginning at six weeks of age. After detection of a vaginal plug, dams were individually housed. Mice were weighed on embryonic day 7 (E7) and embryonic day 10 (E10) with a 1.5g increase in weight indicating pregnancy^40,41^. At E10 pregnant mice were randomly assigned through block randomization to treatment groups and began receiving subcutaneous injections of 10 mg/kg/day fluoxetine in sterile saline or an equivalent volume of sterile saline as a control. Treatments continued through pregnancy and birth (embryonic day 18, E18) and until weaning at lactation on day 21 (L21). Animals were excluded for loss of pregnancy or litter. Mice were ordered, bred, and enrolled in two cohorts to support study feasibility in coordination with vivarium resources (Fig. 5).

#### i Cohort one

Cohort one mice were ordered, bred and enrolled into the experiment in September-December of 2021. Stool samples were collected from dams using sterile tweezers on embryonic day 10 (E10), embryonic day 17 (E17), and lactation day 11 (L11). At lactation day 11, a male and female pup from each litter were sacrificed and stool was collected from the gut using sterile utensils. Stool collection from the gut of L11 pups proved challenging due to small size of the animals and therefore, we piloted two collection procedures including (1) collection of an intact gut segment (containing stool) for extraction and (2) sectioning and scraping the gut interior for stool. Prior to collection, equipment was cleaned with a laboratory-grade detergent and sterilized with 70% ethanol. Bedding used in the bedding transfer procedure for Cohort one was collected and stored at − 80 °C and later introduced to the environment of Cohort two upon arrival. All stool samples collected in this cohort were immediately placed into microcentrifuge tubes and stored at − 80 °C.

#### ii Cohort two

Cohort two mice were ordered, bred and enrolled into the experiment in March–June of 2022. Stool samples were collected from dams using sterile tweezers on embryonic day 10 (E10), embryonic day 17 (E17), lactation day 15 (L15) and lactation day 21 (L21). From the offspring, stool samples were collected on lactation day 15 (L15) and lactation day (21). Samples were not collected from offspring at L11 due to the infrequency and unpredictability of stool sample availability at L11 in Cohort one. Instead, we collected a pooled stool sample from all offspring in each litter at L15. Samples were collected by placing pups on sterile gauze and collecting stool with a sterile tweezers. On L21, a sample was collected from a male and female pup from each litter. All stool samples collected were immediately placed into microcentrifuge tubes and stored at − 80 °C.

### DNA extraction and sequencing

All samples were thawed to room temperature and processed individually. A total of 0.03–0.05g of stool was used for each extraction. Total genomic DNA was obtained by using a bead-beating protocol combined with a phenol:chloroform extraction with the following modification: all aqueous phase washes used 25:24:1 phenol:chloroform:isoamyl alcohol instead of phenol:chloroform for a total of three washes^42^. Four extraction controls were processed with the stool samples and one control was processed during the final PCR reactions.

DNA was quantified using a Qubit fluorometer reagents (Invitrogen, Waltham, MA) and a Synergy 2 microplate reader (BioTek, Winooski, VT, USA). The V4 region of the 16S rRNA gene was amplified via polymerase chain reaction (PCR) using a universal bacterial primer (F-GTGCCAGCMGCCGCGGTAA; R-GGACTACHVGGGTWTCTAAT). Each of these primers were barcoded with individual custom indices to facilitate demultiplexing, as previously described^43^. Each PCR reaction consisted of 12.5 μl KAPA 2 × HiFi Master Mix (KAPA Biosystems, Wilmington, MA, USA), 0.5 μl of 10 μM forward primer, 0.5 μl of 10 μM reverse primer and up to 11.5 μl of 10ng/μl DNA to a total volume of 25 μl with nuclease-free water (IDT, Coralville, Iowa, USA). PCR was conducted on a C1000 Touch™ thermal cycler (Bio-Rad Laboratories, Hercules, CA, USA) with the following amplification protocol: 95 °C for 3 min, 35 cycles of 95° for 30 s, 55 °C for 30 s, and 72 °C for 30 s, followed by a final extension at 72 °C for 5 min. PCR products were quantified on a 1% (w/v) low-melt agarose gel using AquaPor low-melt agarose (National Diagnositcs, Atlanta, GA) using SYBRSafe DNA gel stain (Invitrogen, Waltham, CA), with bands at ~ 380 bp indicating successful amplification. These bands were excised, extracted, and purified using a Zymoclean Gel DNA Recovery Kit (Zymo Research, Irving, CA). A no-template negative control was included with each set of PCRs and if a band was present in the negative control, all samples in that set were redone starting with PCR set-up and amplification. Negative controls for which no band was present had the approximate location of the amplicon (~ 380 bp) excised and sequenced as further confirmation that no contamination was present. The gel-extracted DNA was then quantified using a Qubit fluorometer and a 96-well plate spectrophotometer, and a library was created using a 4 nmol/L equimolar pool of all PCR products. This library was then sequenced on an Illumina MiSeq (Illumina Inc., San Diego, CA) following standard sequencing protocols using a MiSeq v2 2 × 250 bp sequencing kit. Raw sequences from this study were deposited into the National Center for Biotechnological Information’s (NCBI) Short Read Archive (SRA) and are publicly accessible under accession #PRJNA1084828.

### Statistical analysis

Bioinformatic processing and statistical analysis of sequencing reads was performed using QIIME II (2023.5) and R (v 4.1.2)^44,45^. The standard operating procedure described in the QIIME II “Moving Pictures” tutorial, accessed on June 2, 2023^46^, was used to process all sequences. Briefly, paired-end demultiplexed sequences with quality scores in the Casava 1.8 format were imported into QIIME II and the q2-dada2 (v.1.18.0) plugin was used for quality control, trimming, and denoising^47^. A feature table of amplicon sequence variants (ASVs), which are unique sequences not grouped by percentage threshold, was generated. ASVs were aligned with MAFFT (v.7.475), and a phylogenetic tree was constructed using FastTree (v.2.1.1). Taxonomy was assigned using the Silva database (v.138.1, released August 27, 2020) and the classify-sklearn naïve Bayes taxonomy classifier (v0.24.1)^48,49^. The resulting feature table and phylogenetic tree were imported into R for downstream statistical analyses.

The Qiime II feature table and experimental metadata (animal number, treatment, etc.; Microsoft Excel v.2310) were imported into R (1.38.0)^50^. Possible contaminants (Mitochondria, Chloroplasts, Eukaryotes, unclassified) were pruned and ASVs prevalent in negative controls and low read samples (< 5000) were removed with decontam (v.1.14.0)^25,50^. Sequences were rarefied to an even depth of 8000 reads per sample^50^. Alpha diversity was calculated using Shannon’s alpha diversity index and using the Shapiro Wilk test, values were found to have a non-normal distribution. Subsequently, inferential comparisons were made using non-parametric Wilcoxon Signed Rank tests. Beta diversity was visualized using non-metric multidimensional scaling (NMDS) with Bray–Curtis and Unifrac (weighted/unweighted) distance matrixes and inferential comparisons were made using PERMANOVA in the phyloseq package^50^. Testing for differentially abundant taxa was performed using ANCOM-BC (v.1.4.0), a linear regression based method designed to correct biases introduced through differences in sampling fraction^51^. The reported *p* values were adjusted for false discovery rate using parameters internal to ANCOM-BC^51^. For the purposes of this study, results were considered differentially abundant at an (adjusted) alpha level of 0.1.

### Supplementary Information

Supplementary Information.

## Data Availability

The datasets generated during and/or analysed during the current study are available in the National Center for Biotechnological Information’s (NCBI) Short Read Archive (SRA) and are publicly accessaible under accession #PRJNA1084828 at https://www.ncbi.nlm.nih.gov/sra.
