# Supplementary material for: Investigating the influence of perinatal fluoxetine exposure on murine gut microbial communities during pregnancy and lactation
Source: Sci Rep. 2024 Jun 14;14:13762. doi: 10.1038/s41598-024-62224-7 (PMC11178873; doi:10.1038/s41598-024-62224-7)
Supplement: Supplementary file 1 — Supplementary Information. [file 41598_2024_62224_MOESM1_ESM.docx]

Supplementary Information

**S1.** **Offspring Beta Diversity at Lactation Day 11**

Figure Caption: Beta Diversity calculated with Unweighted Unifrac (*p*=0.27), Weighted Unifrac (*p*=0.01*) and Bray Curtis (p=0.02*) distance. Data were visualized through non-metric multidimensional scaling (NMDS) at lactation day 11. Associated values were calculated with PERMANOVA . *denotes statistical significance
